# Ureteral Diverticulum in 65-Year-Old Female

**DOI:** 10.7759/cureus.17310

**Published:** 2021-08-19

**Authors:** Victor Abdullatif, Michael Consolo

**Affiliations:** 1 College of Osteopathic Medicine, Western University of Health Sciences, Pomona, USA; 2 Urology, Inland Urology Medical Group, Pomona, USA; 3 Research, Western University of Health Sciences, Pomona, USA

**Keywords:** ureteral diverticulum, retrograde pyelogram, renal cyst, uti, ureteral malformation

## Abstract

Congenital diverticula of the ureter are a rare class of urologic malformations with very few cases published to date. Here, we present the case of a 65-year-old female who was referred to our clinic with recurrent urinary tract infections. CT urogram demonstrated an apparent left ureteral diverticulum measuring 1.4 x 0.9 cm which was poorly characterized. Subsequent retrograde pyelogram and ureteroscopy confirmed the presence of a ureteral diverticulum (UD) in the left distal ureter.

## Introduction

A ureteral diverticulum (UD) comprises a saccular enlargement of the ureteral wall and is a rare anomaly. There are very few cases reported in the literature. UD varies in terms of localization, size, and presentation. Some patients are asymptomatic while others present with painless hematuria or urinary tract infections. UD has been shown to occur in one of three varieties: congenital, acquired, and abortive. When the UD is a true diverticulum comprising all ureteral layers, it is termed congenital [1]. Acquired diverticula are the result of distal obstruction causing herniation of the mucosa. These are most often caused by calculi, strictures, ureteral valves, or surgical complications. The most frequently reported type of UD in the literature is the abortive diverticulum that results from faulty ureteral budding during embryogenesis [2,3]. We report a case of a 65-year-old female with a ureteral diverticulum who presented with recurrent urinary tract infections.

## Case presentation

A 65-year-old female was referred to our clinic for evaluation of recurrent urinary tract infections (UTIs). She reported approximately four UTIs in the last six months which responded well to antibiotics. She denied fever, hematuria, other pain, or baseline lower urinary tract symptoms. Her family history was non-contributory. Her past medical history was significant for type 2 diabetes mellitus. Her past surgical history included hysterectomy and oophorectomy. Her physical examination was significant for a grade 1 cystocele with evidence of atrophic vaginitis but otherwise unremarkable.

**Figure 1:**
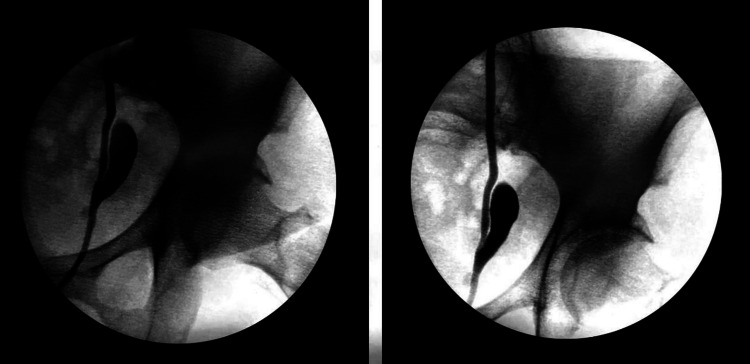
Retrograde pyelogram demonstrating distal left ureteral diverticulum.

**Figure 2:**
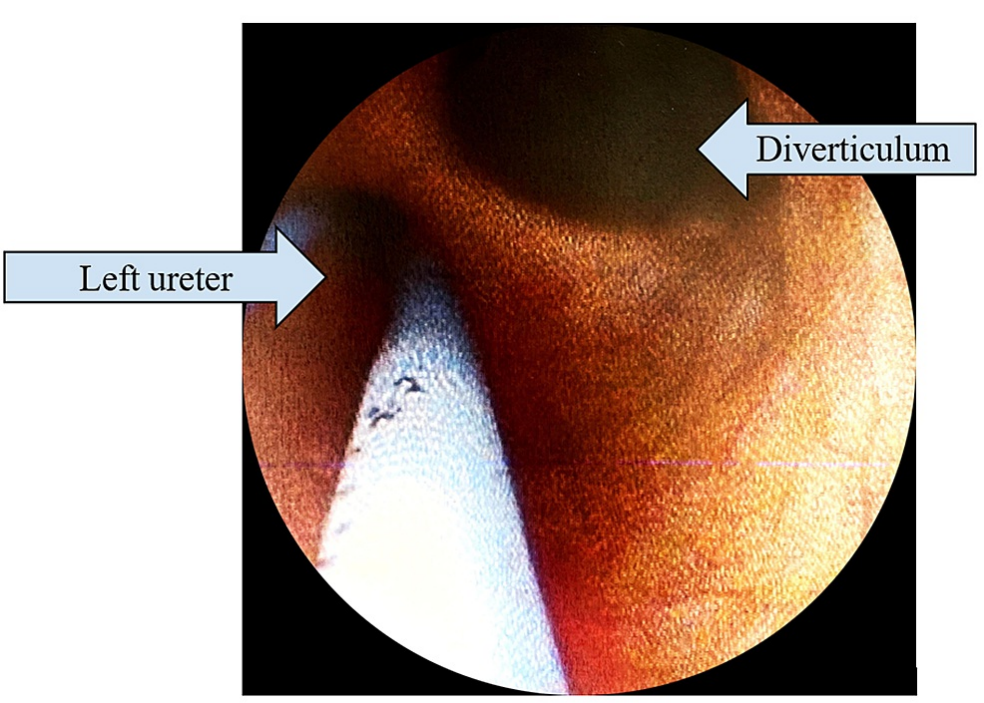
Photograph obtained during ureteroscopy demonstrating ureteral diverticulum.

As the initial part of the work-up, a renal ultrasound was obtained which demonstrated small bilateral simple-appearing cysts and otherwise normal-appearing kidneys. A follow-up CT urogram ordered by her primary physician showed a focal ureteral diverticulum measuring 1.4 x 0.9 cm in the distal left ureter approximately 5 cm proximal to the ureterovesical junction with incomplete opacification on delayed images. Bilateral renal cysts appeared simple and unchanged. No hydronephrosis, filling defects, or areas of ureteral narrowing were discernible. The patient was subsequently brought to the operating room for retrograde pyelogram and diagnostic ureteroscopy. Retrograde pyelogram demonstrated no filling defects but drainage of contrast from the diverticulum was noted to be slightly delayed (Figure 1). The diverticulum was examined ureteroscopically which demonstrated normal-appearing mucosa without evidence of tumor or stricture (Figure 2). The diverticulum measured approximately 2.5 x 1 cm. The remainder of the ureter and collecting system were normal. A double-J stent was left in place for five days and the patient’s postoperative course was uncomplicated. In regard to her recurrent UTIs, the patient opted for vaginal estrogen cream as treatment for atrophic vaginitis but declined any immediate surgical intervention for the diverticulum. She also declined additional medical therapy including antimicrobial prophylaxis.

## Discussion

UD is a rare malformation that has largely been addressed in case reports. The first case of UD was found incidentally during an autopsy in 1808 [4]. A case series involving 12 patients with UD was presented by Barrett and coworkers in 1975 [5]. It has been suggested by Linke and Mongiat-Artus that a UD occurs in ureteral walls at weak areas with incomplete muscle fusion due to the weakness of embryological junctions [6]. It is unclear whether our patient's UD is congenital or acquired.

Women are more likely than men to develop ureteral diverticula. UD are usually left-sided and asymptomatic but may present with pyuria or recurrent UTIs [7]. UD is principally diagnosed via excretory urography. The urogram frequently demonstrates constriction in the ureter and a sharply outlined saccular or globular accessory cavern contiguous with the ureter. On delayed images, a higher rate of contrast accumulation in the cavity of the UD can be observed and the UD opacification remains after the contrast is discharged from the upper urinary tract. The treatment of UD varies depending upon the clinical presentation [1]. Conservative management is preferred in the case of most asymptomatic UDs. If the UD is symptomatic, associated with malignancy, or resulting in complications such as stone formation, compromise of renal function, or infection, intervention is usually necessary [8].

UD treatment should be focused on relieving ureteral obstruction caused by the diverticulum in order to preserve renal function and ensure ureteral patency [1,9]. The surgical methods that have been used in the management of ureteral diverticula include nephrectomy with partial ureterectomy, laparoscopic excision, robotic resection with ureterourethral anastomosis, and suture plication [10]. Some patients with ureteral diverticula have also been found to have associated urothelial malignancies. Wasserman et al. reported an associated bladder tumor present in 26% of their patients with UD [11]. A 2- to 10-year latency in the identification of the diverticula and the development of the tumor was found [9]. Herndon and McKenna discovered a proximal UD antenatally that was aneurysmal and dilated in its entirety [9]. The diagnosis was confirmed in the operating room and, although asymptomatic, was managed with pyeloplasty. A universal approach, they argued, is not practical for the treatment of UD as a result of the limited cases in literature [9]. A more recent study indicates that interventions for UD should only be undergone if the UD is complicated by infection, stone formation, decreased renal function, or malignancy. In said study, a female patient presented with 6 months of left loin pain. Left-sided proximal hydroureteronephrosis was found on CT and a diverticulum was revealed on retrograde ureterography. This patient underwent robot-assisted diverticulectomy and ureteral anastomosis [1].

This case report highlights the importance of contrast-enhanced CT with delayed images (CT urogram) in certain cases of complicated recurrent UTIs as renal ultrasound and/or non-contrast CT scans can miss certain rare or unusual pathologies. Although our findings suggest that a CT urogram may play an important role in uncovering congenital or acquired ureteral diverticula, we do not suggest that a CT urogram is required in all cases of recurrent UTIs as ureteral diverticula are an exceptionally rare class of malformations.

## Conclusions

Our patient presented with an uncomplicated UD and is not willing to undergo surgical management at the time of writing. She will undergo continued surveillance for UTIs. Operative management will be considered if the patient fails conservative management or if she experiences complications.
